# Supplementary material for: Alcohol Participates in the Synthesis of Functionalized Coumarin-Fused Pyrazolo[3,4-b]Pyridine from a One-Pot Three-Component Reaction
Source: Molecules. 2019 Aug 4;24(15):2835. doi: 10.3390/molecules24152835 (PMC6696490; doi:10.3390/molecules24152835)

Alcohol Participates in the Synthesis of Functionalized Coumarin-Fused Pyrazolo[3,4-*b*]Pyridine from a One-Pot Three-Component Reaction

Wei Lin [1,*], Cangwei Zhuang [1], Xiuxiu Hu [1], Juanjuan Zhang [2] and Juxian Wang [3,*]

[1] School of Chemistry and Environmental Engineering, Jiangsu University of Technology, Changzhou 213001, China
[2] State Key Laboratory of Pharmaceutical Biotechnology, Nanjing University, Nanjing 210093, China
[3] Institute of Medicinal Biotechnology, Chinese Academy of Medical Science and Peking Union Medical College, Beijing 100050, China
* Correspondence: linwei@jsut.edu.cn (W.L.); imbjxwang@163.com (J.-X.W.)

Academic Editor: Derek J. McPhee and Roman Dembinski

Received: 10 July 2019; Accepted: 2 August 2019; Published: 4 August 2019

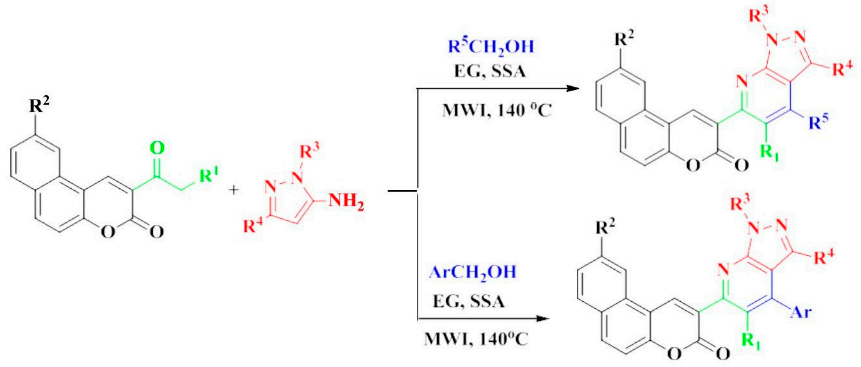

Supplement: Supplementary file 1 [file molecules-24-02835-s001.pdf]
